# “Penguins don't fly”: An investigation into the effect of typicality on picture naming in people with aphasia

**DOI:** 10.1080/02687038.2012.751579

**Published:** 2013-02-19

**Authors:** Clare Rossiter, Wendy Best

**Affiliations:** 1 Whipps Cross University Hospital, London, UK; 2 Psychology and Language Sciences, University College London, London, UK

**Keywords:** Naming, Anomia, Typicality

## Abstract

**Background:**

Previous research has highlighted psycholinguistic variables influencing naming ability for individuals with aphasia, including: familiarity, frequency, age of acquisition, imageability, operativity, and length (Nickels & Howard, 1995) and a potential link between typicality and generalisation to untreated items in intervention (Kiran, Sandberg, & Sebastian, 2011). However, the effect of concept typicality (the extent to which an item can be considered a prototype of a category) on naming in aphasia warrants further examination.

**Aims:**

To investigate first whether typicality can be reliably rated across a range of natural semantic categories and second whether, and if so in which direction, typicality influences naming performance for people with aphasia. To provide quantitative and qualitative information on typicality for a set of stimuli for use in future research.

**Methods & Procedures:**

Typicality ratings were obtained and the results compared with those in the existing literature. The influence of typicality on picture naming was investigated employing both matched sets (high and low typicality matched for other psycholinguistic variables) and logistic regression analyses for the group and individual participants with aphasia (*n* = 20).

**Outcomes & Results:**

Typicality rating correlated strongly with ratings obtained in previous research (Rosch, 1975: *r* = .798, *N* = 35, *p* < .001; Uyeda & Mandler, 1980: *r* = .844, *N* = 47, *p* < .001). Typicality was a significant predictor of picture naming for the group and some individuals, with generally better performance for typical items. This was demonstrated in both matched sets and regression analyses. However, other psycholinguistic variables proved more strongly related to naming success, particularly age of acquisition.

**Conclusions:**

Typicality can be rated reliably and should be considered alongside other psycholinguistic variables when investigating word retrieval and intervention in aphasia. Further research is necessary to accurately model the direction of typicality effects found in word retrieval. Finally, the differing nature, size, and internal structure of categories require further exploration when investigating typicality effects.

Extensive research has investigated the factors contributing to naming disorders in aphasia. Several variables have been shown to influence lexical access, both for people with unimpaired language-processing skills and individuals with naming disorders, including: frequency, familiarity, imageability, concreteness, length, and age of acquisition (Ellis, Lum, & Lambon Ralph, 1996; Nickels, 1997). Variables predicting naming accuracy are frequently intercorrelated (Feyereisen, Van der Brought, & Seron, 1988; Morrison, Ellis, & Quinlan, 1992; Woollams, 2012).

Research in the field of semantic memory suggests that concept typicality (how closely the features and characteristics of an item match the prototype of a category) is an important component of an item's semantic representation. For example, a *robin* could be considered typical of the category *bird* (e.g., flies, small, has wings, feathers, beak, builds nests) whereas a *penguin*, though still a bird, has fewer prototypical features (e.g., large) with some particularly uncharacteristic, distinctive attributes (e.g., cannot fly) and may therefore be considered a less typical exemplar of its category.

Several models of semantic memory for concrete concepts have been proposed, including spreading activation (Collins & Loftus, 1975) and feature-based prototype theories (Rosch & Mervis, 1975; Smith, Shoben, & Rips, 1974). More recently, connectionist computer simulations have also been used to predict and investigate effects (Plaut, 1996; Rogers et al., 2004). Through connectionist modelling techniques, the concept of a multi-dimensional semantic space has been proposed with typical items occupying a central area with a greater number of shared, overlapping features and atypical items, with idiosyncratic features, found on the periphery.

Typicality has been shown to influence performance in individuals with unimpaired language-processing skills with faster responses in category verification tasks for high than low typicality items (Kiran, Ntourou, & Eubanks, 2007; Kiran & Thompson, 2003b; Larochelle & Pinneau, 1994; Rosch, 1973; Smith et al., 1975).

A preferential effect for high typicality items has also been found in semantic dementia (Woollams, 2012; Woollams, Cooper-Pye, Hodges & Patterson, 2008). For the purpose of the current study it should be noted that, as semantic dementia is a progressive degenerative disease, research findings cannot be viewed as directly comparable to aphasia, as the semantic deficits arise from two different aetiologies (Jefferies & Lambon Ralph, 2006; Jefferies, Rogers, Hopper, & Lambon Ralph, 2010). However, the research includes a large number of participants with semantic dementia in a case series design and has provided valuable insights regarding typicality effects and how these may inform models of semantic memory.

Woollams and colleagues (Woollams, 2012; Woollams et al., 2008) observed an appreciable typicality effect in picture-naming performance for people with semantic dementia, most significant for those at the moderate stage, demonstrating better-preserved naming for higher typicality items and poor performance for atypical items. Typicality was found to be strongly correlated with other variables, particularly age of acquisition and frequency. When these correlated variables were controlled, a significant effect was demonstrated for both age of acquisition and frequency, but not typicality. However a significant interaction between typicality and severity remained. Error analysis indicated participants frequently gave higher typicality responses for atypical items. This pattern has been shown in other tasks for people with semantic dementia, including delayed copy drawing where unusual atypical features are omitted and often replaced with more typical features (Bozeat et al., 2003). Woollams and colleagues (Woollams, 2012; Woollams et al., 2008) therefore propose that lower typicality items with fewer intercorrelated features are likely to be more susceptible to damage for individuals with a deficit in semantic memory. This result has been replicated in a connectionist model of semantic memory (Rogers et al., 2004).

This typicality effect was replicated for unimpaired participants by applying repetitive transcranial magnetic stimulation (rTMS) to the left anterior temporal lobe, a primary locus of deficit in semantic dementia (Woollams, 2012). Results showed poorer performance for atypical items, supporting the prediction that lower typicality items would be more vulnerable following damage in this area. Woollams argues that this lends weight to the theory that semantic representations are stored in an amodal hub in the anterior temporal lobes which connect with different modality-specific featural representations as part of a “hub and spoke” model of semantic processing (Patterson, Nestor, & Rogers, 2007).

The influence of typicality on the performance of people with aphasia has also been investigated. Kiran and Thompson (2003b) found a typicality effect for participants with non-fluent aphasia in category verification tasks using animate categories, with typical items processed faster and more accurately. However, the predicted typicality effects were not seen for participants with fluent aphasia. A study carried out by Kiran et al. (2007) using inanimate categories in a verification task also found this typicality effect, but those participants with aphasia assigned to the semantic impairment group demonstrated reduced accuracy rates for both typical and atypical items.

Exploring the effects of typicality on word retrieval for people with aphasia is important because anomia treatment studies (Kiran, 2008; Kiran & Thompson, 2003a) have shown generalisation for naming untreated typical items within a natural category when participants were treated using atypical items (see also Kiran et al., 2011). This contrasts with the more usual item-specific treatment effects (Nickels, 2002). However, the studies showing generalisation examined a relatively limited number of items and semantic categories.

The findings from aphasia intervention studies have been related to connectionist modelling of typicality effects. In order to examine the effects of relearning after damage, Plaut (1996) trained, lesioned and retrained a connectionist simulation model of semantics. The network learnt typical items better than atypical words during initial retraining. However, Plaut's study found retraining the lesioned computer network using *atypical* items resulted in generalisation to untreated *typical* items. While retraining using *typical* items resulted in improved naming for other *typical* items, results showed no generalisation and, notably, deterioration in naming performance for *untreated atypical* items. This model has therefore been used to support findings from recent intervention studies with adults with aphasia (Kiran, 2008; Kiran & Thompson, 2003a).

Notably in relation to the current study, the initial lesions in Plaut's model, prior to retraining, showed substantially more impaired performance for typical words than for atypical words. To explain this finding Plaut suggests it is easier for the network to distinguish between atypical words which have fewer close neighbours due to their distinguishing features. The direction of this effect contrasts with predictions and modelling in semantic dementia (Rogers et al., 2004; Woollams, 2012). However these models are not directly comparable, as the model developed by Rogers et al. was lesioned on a step-by-step basis to reflect the progressive nature of semantic dementia and the modelling covered a wide range of semantic tasks.

A final consideration when investigating concept typicality is the effect of the varied nature, size, and internal structure of semantic categories. This is important, as research relating to typicality has demonstrated differences between animate and inanimate categories (Garrard, Lambon Ralph, Hodges, & Patterson, 2001) and natural versus well-defined categories (Kiran & Johnson, 2008).

This report therefore asks whether typicality can be reliably rated across a wide range of different semantic category types. Ratings from the current study will be investigated to determine if correlations exist with typicality ratings collected from studies several decades ago. The current study obtains typicality data from a population which is varied for age, gender, and educational background, as some earlier studies have tended to collect data from a more specific group (e.g., psychology undergraduate students). In addition, qualitative data will be obtained to support the rating given, with the aim of highlighting which attributes or features are considered most salient in determining typicality, in order to provide stimuli lists and typicality ratings that may be of use in future research. As previous intervention studies have tended to focus on a relatively small number of semantic categories, further information regarding typicality across a broad range of items to investigate for possible differences depending on the internal structure of a category would be beneficial. Finally, the effect of typicality on picture-naming performance for people with aphasia is investigated, examining the strength of any typicality effect compared to the effect of other psycholinguistic variables and relating findings to earlier research and predictions based on computational modelling.

## METHOD

### Participants

*Raters.* Data were collected from 32 participants to obtain typicality ratings for each individual. All participants (16 men and 16 women) had English as a first language. Participants ranged in age from 22 to 64 years and half had a University degree

*Participants with aphasia.* Data from 20 people with acquired aphasia with English as a first language were used in this study. All participants (11 men and 9 women) were at least 1 year post-stroke and reflected a heterogeneous population of people with aphasia, comprising 8 fluent and 12 non-fluent speakers (Table 1). Fluency was determined following assessment by a speech and language therapist.

**TABLE 1:** Background information for participants with aphasia

	*Age*	*Gender*	*Aphasia type*	*Years post-onset*
DA	58	Male	Non-fluent	1
BB	50	Male	Non-fluent	1
DC	70	Female	Fluent	4
SC	65	Male	Fluent	4
PH	77	Female	Fluent	2
LJ	64	Male	Non-fluent	4
IK	68	Male	Non-fluent	4
NK	52	Male	Fluent	4
OL	65	Female	Fluent	2
HM	45	Male	Non-fluent	5
QP	65	Male	Non-fluent	5
KR	38	Female	Non-fluent	12
FA	64	Female	Non-fluent	2
GB	71	Male	Non-fluent	3
TE	69	Male	Fluent	1
DJ	65	Female	Fluent	1
CM	52	Male	Non-fluent	5
LM	42	Female	Non-fluent	7
PP	75	Female	Fluent	2
CV	56	Female	Non-fluent	2

Pre-therapy language assessment results for individual participants with aphasia are shown in Table 2. Performance on Comprehensive Aphasia Test spoken word to picture matching ranged from 67% to 100%. Semantic assessment using Pyramids and Palm Trees three-picture version ranged from 42% to 100%. Phonological assessment, measured by real-word repetition ranged from 31% to 99% correct.

**TABLE 2:** Pre-therapy background assessment data for participants with aphasia

	*CAT Spoken Word to Picture Matching: Percentage correct*	*CAT Written Word to Picture Matching: Percentage correct*	*Pyramids and Palm Trees. Three-picture version: Percentage correct*	*Repetition of real words (n = 152): Percentage Correct*
DA	0.90	0.87	0.94	0.31
BB	0.97	1.00	0.92	0.82
DC	1.00	0.97	0.92	0.95
SC	0.87	0.77	0.88	0.57
PH	0.93	0.97	0.90	0.97
LJ	0.77	0.40	0.42	0.96
IK	0.93	0.80	0.92	0.52
NK	0.93	0.97	0.87	0.99
OL	0.97	0.93	0.96	0.99
HM	1.00	0.87	0.94	0.73
QP	0.90	0.97	0.88	0.90
KR	0.93	0.90	0.77	0.90
FA	0.87	0.90	0.79	0.36
GB	0.87	0.90	0.94	0.36
TE	1.00	1.00	1.00	0.87
DJ	0.97	0.97	0.96	0.45
CM	0.83	0.90	0.94	0.70
LM	0.97	1.00	0.92	1.00
PP	0.87	0.97	0.83	0.57
CV	0.67	0.73	0.65	0.89

Comprehensive Aphasia Test (Swinburn, Porter, & Howard, 2005); Pyramids and Palm Trees (Howard & Patterson, 1992), and 152 real words (Howard, personal communication).

### Stimuli

A total of 200 black-and-white line drawings of objects, including both living (e.g., animals, vegetables) and non-living items (e.g., furniture, tools), were assigned to categories by the authors, referring where applicable to earlier typicality studies (Rosch, 1975; Uyeda & Mandler, 1980) and Battig and Montague's (1969) category norms tables. All drawings had 95% naming agreement among controls with unimpaired language. Seven speech and language therapists then carried out a category verification task. Items with agreement of 5/7 or above (*n* = 172) were rated for typicality. Following the rating task, a further five items were removed, as raters had questioned the validity of the category label assigned. The naming data obtained from the participants with aphasia were analysed for the remaining 167 items.

### Procedure

*Ratings.* Raters were asked to provide a quantitative rating of how typical they considered each of the items to be of the given category, using a 7-point rating scale, where 1 = typical, 7 = least typical, and 4 = moderate fit. This method replicated earlier studies measuring typicality (Kiran & Thompson, 2003a; Rosch, 1975). In addition, raters were asked to give qualitative information to support the numerical rating given; further details are provided in Supplementary Material 1 (available via the supplementary tab on the article's online page at http://dx.doi.org/10.1080/02687038.2012.751579).

*Picture-naming assessment.* Data used in the current study were obtained from pre-therapy baseline measures as part of two wider therapy studies (Best, Greenwood, Grassly, & Hickin, 2008; Hickin, Best, Herbert, Howard, & Osborne, 2002). Each participant carried out a confrontation picture-naming assessment for 200 items on two occasions, at least 8 weeks apart.

*Data analysis.* The naming data were analysed in two ways in order to seek converging evidence from different methods (Ellis et al., 1996). In view of the heterogeneity and variability within aphasia, and possible resultant difficulties interpreting group effects, previous research emphasises the importance of analysing findings for individual participants within a group design (Nickels & Howard, 1995) and more recently for case series (Schwartz & Dell, 2010).

Matched sets, including *t*-values (indicating the difference between high “HT” and low “LT” typicality sets) for each variable: typicality (Typ.), familiarity (Fam.), imageability (Image.), concreteness (Conc.), age of acquisition (AoA.), operativity (Oper.), frequency (Freq.), lemma frequency (Lemma Freq.), number of syllables (Syll.), and number of phonemes (Phon.).

*Matched sets.* Using the mean typicality ratings, 39 items were assigned to a high typicality set and 39 items to a low typicality set. Mean scores for the variables of familiarity, imageability, concreteness, age of acquisition, operativity, length, and frequency were closely matched between the sets^1^ (Table 3; full details are provided in Supplementary Material 2). Naming performance on the sets could then be compared, to investigate any typicality effect on naming for people with aphasia, while controlling for possible confounding variables.

**TABLE 3:** Matched sets

	*Typ.*	*Fam.*	*Image.*	*Conc.*	*AoA*	*Oper.*	*Freq.*	*Lemma freq.*	*Syll.*	*Phon.*
HT set mean	1.55	533.74	599.87	590.46	2.65	4.27	1.04	1.27	1.82	4.85
Standard deviation	.21	53.12	28.84	31.30	.65	.81	.67	.59	.79	1.71
LT set mean	4.05	520.15	596.82	596.26	2.75	3.88	1.05	1.22	1.69	4.49
Standard deviation	0.80	59.16	29.35	22.47	0.59	0.87	0.48	0.46	0.73	1.47
Difference between means	−2.51	13.59	3.05	−5.79	−.10	.40	.00	.05	0.13	0.36
*t*	−10.16	.25	.10	−.18	−.15	.48	−.01	.08	.16	.21

*Logistic regression.* Howard, Best, Bruce, and Gatehouse (1995) and Cutler (1981) have highlighted limitations of matched sets analysis, including omitting relevant data and small differences in variables between sets, which may contribute to an observed effect. Therefore regression was employed as an additional method to investigate any typicality effect.

## RESULTS

### Can typicality be reliably measured across a range of semantic categories?

Typicality ratings from the current study correlated significantly with those from Rosch (1975) (Pearson *r* = .798, *N* = 35, *p* < .001). A significant correlation was also observed between the current ratings and those obtained by Uyeda and Mandler (1980) (Pearson *r* = .844, *N* = 47, *p* < .001). Information from the qualitative typicality ratings is provided in Supplementary Material 3.

### What correlations exist between typicality and other psycholinguistic variables?

Typicality ratings collected in the current study were entered into a correlation matrix alongside data for the variables of familiarity, imageability, age of acquisition, operativity, frequency, and length. Results demonstrate that typicality was significantly positively correlated with age of acquisition and significantly negatively correlated with familiarity, operativity, and frequency (Table 4).^2^ The full correlation matrix is provided in the Supplementary Material 4.

**TABLE 4:** Variables significantly correlated with typicality (*p* = < .01)

	*Familiarity*	*Age of acquisition*	*Operativity*	*Log Lemma frequency*
Typicality	−.372	.176	−.246	−.266

### Does typicality influence naming ability for people with aphasia?

*Matched sets analysis.* Total scores correctly named for high (HT; *N* = 39) and low (LT; *N* = 39) typicality sets were collected for each participant with aphasia. For the group there was a significant difference between the sets with superior naming of the high typicality set (HT Set Mean = 38.60 LT Set Mean = 34.65), paired sample *t*-test *t*(19) = 3.061, *p* = .006, two-tailed, *d* = .702.

**Figure 1.**
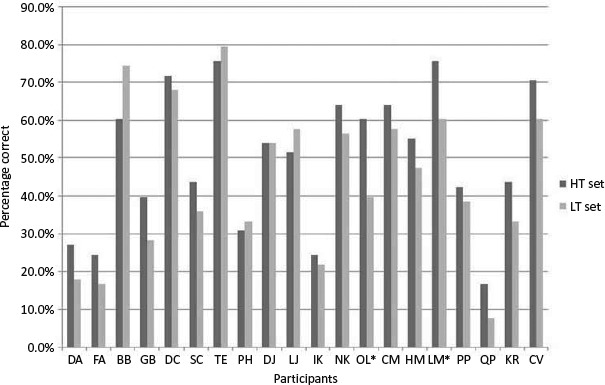
Percentage of correct responses across both naming attempts (out of 78 items) for high and low typicality sets.

Figure 1 illustrates the percentage of high and low typicality items correctly named for each individual participant with aphasia: 15 participants correctly named a higher number of items in the high typicality set; 4 individuals demonstrated the opposite trend, correctly naming a higher percentage in the low typicality set. For one participant there was no difference between the sets.

In order to investigate typicality effects for individual participants, each item (*N* = 78, grouped into the HT and LT sets) was given a combined score corresponding to the number of times correctly named across both pre-therapy assessments (e.g., 0 = not named on either occasion, 1 = named on one occasion, 2 = named on both occasions). Using a one-tailed hypothesis that individuals would show preferential naming for the HT set, based on findings from previous studies with people with aphasia, two participants showed a significant difference between conditions (independent *t*-test; OL: *p* = .049; LM: *p* = .026).

*Regression analysis.* Generalised estimating equations (GEEs) were used to investigate the effect of typicality on naming for the group. GEEs can model a binary response when the data are not independent (as in this study) because the naming responses by each participant constitute repeated measures. This method incorporates other independent variables into the regression model, enabling investigation of their relative strength as naming predictors (e.g., estimating the increase or decrease in the odds of getting a correct or incorrect response).

To prevent possible suppression effects arising from the inclusion of correlated predictor variables, concreteness (which correlated with imageability) and familiarity (which correlated with frequency) were omitted from the regression model. The following were entered: typicality, frequency, age of acquisition, operativity, and word length (number of phonemes).

The group regression analysis showed typicality as a significant predictor of naming ability (*p* = .036) with higher typicality items decreasing the probability of producing an error response. However, results also show that typicality is a relatively weak predictor, when compared to other independent variables, including age of acquisition, word length, and operativity, which are highly significant naming predictors (Table 5).

**TABLE 5:** Group regression analysis: dsemonstrating the significance of each independent variable for the group as a whole

*Variable*	*Odds ratio*	p *value*
Typicality	1.049	.0359
Imageability	1.003	.0012
Age of acquisition	1.507	<.0001
Operativity	1.206	<.0001
Frequency	1.209	.0052
Word length	1.182	<.0001

There was also an effect of Time (Odds ratio 1.135, *p* = .0396) resulting from some participants demonstrating better picture naming on the second occasion of testing. However, this is not a focus of the current investigation; see Hickin et al. (2002) and Best et al. (2008) for details.

Logistic regression analyses were also carried out for individual participants. When entered into the regression model as a single variable, typicality had a significant effect on naming for five participants: DJ, NK, OL, PP, and KR (Table 6). “Percentage Model” refers to the percentage of cases (correct vs incorrect responses) correctly predicted by the model. However, significant Hosmer-Lemeshow tests for two participants (DJ and OL) indicated the model cannot be considered a good fit of the data for these individuals, leaving three for whom there was a clear effect of typicality on naming employing this method at the single case level.

**TABLE 6:** Results demonstrating significant typicality effects for five individuals, with typicality entered as a single variable

*Participant*	*Percentage model(%)*	*Typicality sig.*	*Percentage model including typicality(%)*	*Odds ratio*
DJ	52.1	.005	54	.745
NK	57.9	.007	59.1	.755
OL	51.8	<.0001	57.3	.648
PP	61.3	.005	60.7	.735
KR	61.3	.005	61.3	.730

## DISCUSSION

The results demonstrate that concept typicality can be reliably rated and, along with other psycholinguistic variables, influence naming in people with aphasia.

### Can typicality be reliably rated across a range of semantic categories?

The current study demonstrated high inter-study reliability with previous studies where typicality ratings were collected from psychology undergraduate students (Rosch, 1975; Uyeda & Mandler, 1980). A significant correlation was found, suggesting typicality can be reliably rated as a concept despite the studies spanning a timeframe of over 35 years, originating from different geographical areas and using raters of different ages and levels of education. Further investigation is warranted to consider in more detail whether typicality is processed differently depending on the type, size or nature of the semantic category.

### The relationship between typicality and other psycholinguistic variables

Typicality was significantly correlated with frequency, age of acquisition, operativity, and familiarity. Typicality's strong correlation with familiarity is unsurprising, mirroring previous research (Malt & Smith, 1982; McCloskey, 1980). Exploration of qualitative data from the current study also suggests a relationship between these two variables, as for some highly typical and atypical items the commonality or rarity of an item was given as a reason to support the quantitative typicality rating (see Supplementary Material Qualitative Ratings List 1). The close correlation between typicality and age of acquisition in the current study is also in line with the findings of Woollams (2012).

The significant correlations between typicality and some other psycholinguistic variables suggest these should not be considered in isolation. In future studies typicality should be viewed in the context of its correlations with other variables, particularly familiarity and age of acquisition.

### Does typicality influence naming ability for people with aphasia?

As interpreting results between intercorrelated variables can be problematic, two forms of statistical analysis were used to investigate this question; matched sets and logistic regression.

Using matched sets analysis the group were shown to be significantly better at naming higher typicality items. This is in line with earlier unpublished research (Howard & Best, 1996) where a significant group effect was also found with preferential naming performance for highly typical items. The matched sets data also showed numerically better naming performance for the highly typical set for 15/20 individual participants, with this effect reaching statistical significance for two participants. On the basis of the background language assessments shown in Table 2 there does not appear to be a clear association in terms of the direction of the effect between outcomes for those participants with primarily semantic deficits and those with primarily phonological deficits.

In the regression analysis a significant effect was found for the group as a whole when typicality was entered as a single variable. Typicality remained significant when other independent variables were entered into the model, but was a relatively weak predicting variable (only imageability was a weaker predictor and the picture stimuli are all, by nature, highly imageable).

In individual regression results typicality was a significant predictor of naming for five participants when entered as a single variable. However, for the remaining 15 individuals, typicality did not significantly predict naming success. Notably, age of acquisition proved the most strongly predictive variable of naming success for 10 of the 20 participants. This included four out of the five participants who demonstrated individual typicality effects in the regression analyses. Furthermore, and of concern, the individuals for whom there was a significant typicality effect were not identical across the two methods of analysis (matched sets: OL and LM; logistic regression: DJ, NK, OL, PP, KR). In addition, for DJ and OL the statistical model did not provide a good fit to their data. The different findings suggest that noise in the data is influencing the outcome and highlights the need for very thorough investigation before specific psycholinguistic variables are claimed to influence individual performance.

### Main finding

The most robust finding, consistent across all four analyses, is the direction of the significant typicality effects; better performance for high than low typicality items. The observed effect of typicality on naming may reflect easier access to items sharing many features prototypical to the category, in line with the predictions of Rogers et al. (2004) and the results that Woollams (2012) found in people with semantic dementia. The direction of the typicality effect contrasts with that found by Plaut (1996) in his model following lesioning, but prior to retraining. Plaut focused on reading and semantics employing a model previously used to map from orthography to semantics, while the data in this study are from a picture-naming task. However, the same modelling has been invoked to explain the findings from intervention studies manipulating typicality where the outcome is also naming. Further modelling focused at the level of production would therefore be helpful to provide more information regarding the direction of the effect.

### Limitations of the current study and implications for future research

The current study uses data from participants included in anomia therapy studies which did not focus directly on typicality and in which participants were not matched for deficit type. Future research to investigate the relationship between the nature of the primary impairment in aphasia and any influence of typicality on performance may also be beneficial particularly when linking the findings to implications for intervention. Stanczak, Waters, and Caplan (2006) investigated typicality in an intervention study for two participants with anomia. Results indicated significant generalisation effects to untrained typical items for the participant with both semantic and phonological deficits. However, the participant with phonological difficulties demonstrated faster learning for typical items and did not show generalisation to untrained items. Thus Stanczak et al. (2006) and Kiran and Johnson (2008) highlight type of deficit as an important future consideration for anomia intervention studies.

Detailed analysis regarding aphasia error types produced by participants may also provide valuable information as indicated by Woollams and colleagues (Woollams, 2012; Woollams et al., 2008). However this was not included in the data for the current study and is a limitation.

Possible differences in typicality depending on the type, size, and nature of semantic category is a potentially important factor, which requires further exploration (Garrard et al., 2001; Kiran & Johnson, 2008; Larochelle, Richard, & Soulierres, 2000). Although a full analysis of the qualitative data provided to support the typicality ratings obtained is beyond the scope of the current study, some observations can be made, which may have implications for future research.

Qualitative information for the animate category, *animals* (Supplementary Material: Qualitative Listings 3) appears to support the findings of Garrard et al. (2001) showing a higher ratio of sensory to functional features (e.g., has four legs, fur, tail) which frequently overlapped with other category members. Also as expected, for the inanimate category *kitchen utensils*, reasons given to support typicality ratings tended to refer to an item's function or use (see Supplementary Material: Qualitative Listings 4).

Some issues remain when investigating typicality effects across a broad range of categories, and the qualitative data speak to this issue. For example, some items may overlap and meet membership of more than one category but their typicality rating may vary depending on category choice (e.g., tank = weapon/vehicle). For the purpose of the current study tank was included as a weapon on the basis of the initial category verification task and a higher response rate for Battig and Montague's (1969) word production frequency category norms. However qualitative data indicated that some participants felt this item could also be categorised as a vehicle. If given this categorisation, it is likely that a lower typicality rating would have been provided.

Similarly the inclusion of subordinate categories with a smaller number of exemplars compared to larger subordinate categories might also influence the typicality rating given. Crocodile, judged to be a reptile in the initial category verification task, was given a high typicality rating. However, if placed within the wider superordinate category of *animal*, it is likely to be considered a far more atypical example, as illustrated by the fact that only 3 out of 442 respondents generated this exemplar in Battig and Montague's category norms for four-footed animal.

Finally, the current study includes exemplars from a wide range of natural categories (e.g., *birds*) with less-distinct boundaries and graded representations and a small number of well-defined categories (e.g., *shapes, body parts, occupations*) which tend to have more clear-cut, rigid boundaries in terms of category membership. Kiran and Johnson (2008) have reported equivocal support for typicality effects in an intervention study using well-defined categories, (e.g., shapes) but highlight the abstractness of these categories. Interestingly, a number of exemplars from well-defined categories are included in Qualitative Listings 2 (Supplementary Material) where five or more participants were unable to provide a qualitative reason to support their typicality ratings. The appendices which provide quantitative and qualitative information for typicality ratings may be useful in future research.

Further research regarding internal category structure and the organisation of semantic category representations is required in order to investigate the possibility that typicality might be more tangible, easily applied, and reliably measured in some semantic categories than others. This may be an inherent difficulty when attempting to measure typicality across a wider range of categories and these issues need to be carefully considered in future studies investigating typicality and therapeutic intervention.

## Footnotes

1 Frequency values were obtained from the Celex Database (1993). Operativity ratings were taken from an earlier study (Howard et al., 1995). Data for remaining variables were sourced from the MRC psycholinguistic database (Coltheart, 1981).

2 It should be noted that, in order to replicate earlier studies collecting typicality ratings, a scale of 1 (typical) to 7 (atypical) was used, resulting in a numerically *low* overall rating corresponding to a *highly* typical item, and a numerically *high* rating corresponding to an *atypical* item. Therefore the negative correlations observed indicate that items rated as *highly* typical are generally *higher* in familiarity, operativity, and frequency.
